# Post-marketing surveillance of upadacitinib: multilevel analysis of venous thromboembolism reporting in global data and rheumatoid arthritis

**DOI:** 10.3389/fmed.2025.1683751

**Published:** 2025-12-01

**Authors:** Renato Ferreira-da-Silva, Mariana Lobo, Ana Rafaela Abreu, Juliana Pereira-Macedo, Beatriz Mós, Carolina Ameijeiras Rodriguez, Inês Mariana Lourenço, Isabel Vieira, Jorge Junqueira Polónia, Luís Gouveia, Lurdes Silva, Manuela Morato, Manuela Pinto, Mario Forrester, Marta Pereira, Nuno Rodrigues, Tayanny Biase, Inês Ribeiro

**Affiliations:** 1 Porto Pharmacovigilance Centre, Faculty of Medicine of the University of Porto, Porto, Portugal; 2 RISE-Health, Department of Community Medicine, Information and Health Decision Sciences, Faculty of Medicine of the University of Porto, Porto, Portugal; 3 RISE-Health, Department of Surgery and Physiology, Faculty of Medicine, University of Porto, Porto, Portugal; 4 RISE-Health, Department of Medicine, Faculty of Medicine of the University of Porto, Porto, Portugal; 5 LAQV@REQUIMTE, Laboratory of Pharmacology, Department of Drug Sciences, Faculty of Pharmacy of the University of Porto, Porto, Portugal; 6 Local Health Unit of São João, E.P.E., Porto, Portugal; 7 Local Health Unit of Santo António, E.P.E., Porto, Portugal; 8 Local Health Unit of Vila Nova de Gaia/Espinho, E.P.E., Porto, Portugal

**Keywords:** Janus kinase inhibitors, venous thromboembolism, pharmacovigilance, drug-related side effects and adverse reactions, rheumatoid arthritis, adverse drug reaction reporting systems

## Abstract

**Background:**

Upadacitinib is an oral Janus kinase 1 (JAK1) selective inhibitor approved for the treatment of rheumatoid arthritis (RA) and other immune-mediated inflammatory diseases. Concerns have emerged regarding a potential increased risk of venous thromboembolism (VTE) with JAK inhibitors (JAKi), though real-world evidence remains limited.

**Objective:**

To assess the post-marketing safety profile of upadacitinib in relation to VTE using global pharmacovigilance data.

**Methods:**

We conducted a disproportionality analysis using Individual Case Safety Reports (ICSRs) from VigiBase, accessed via VigiLyze, including all reports up to February 20, 2025. Upadacitinib was compared with: (i) all other medicines; (ii) other second-line advanced RA therapies (bDMARDs and tsDMARDs); and (iii) other JAKi (baricitinib, tofacitinib, filgotinib). Reporting Odds Ratios (ROR) and Information Components (IC), with 95% confidence intervals, were calculated.

**Results:**

Descriptive analyses identified 678 VTE cases with upadacitinib, predominantly affecting women (68.1%), with a median age of 63 years. Most were classified as serious (91.2%), although fatal outcomes were less frequent than with comparators. In disproportionality analyses, upadacitinib showed a significant signal versus all medicines (ROR: 2.08; IC: 1.04) and versus other second-line therapies (ROR: 1.40; IC: 0.41), but not versus other JAKi (ROR: 0.98; IC: –0.04). In 2023, disproportionality declined, particularly relative to other JAKi (ROR: 0.57; IC: –0.38).

**Conclusion:**

Upadacitinib-related VTE cases display distinct clinical characteristics. These findings support continued pharmacovigilance and the need for robust real-world studies to clarify absolute and comparative risks, inform regulation, and guide personalised therapeutic strategies in RA.

## Highlights

- Upadacitinib was associated with a higher-than-expected reporting of venous thromboembolism (VTE) compared with all medicines in VigiBase, other second-line therapies, and other JAK inhibitors (JAKi) for rheumatoid arthritis, suggesting a potential safety signal warranting monitoring in clinical practice.
- Within the JAKi class, upadacitinib showed a nuanced safety profile, with fewer reports of deep vein thrombosis but higher reports of pulmonary thrombosis compared with other JAKi, suggesting event-type-specific differences in VTE risk.
- Age-stratified analyses revealed that upadacitinib had higher VTE reporting in nearly all age groups compared to other JAKi, except in patients aged 18–44 years, highlighting the need for age-specific risk assessment when prescribing upadacitinib.
- In 2023, the disproportionality signal for VTE with upadacitinib attenuated compared to previous years and to other JAKi, potentially reflecting evolving prescribing practices, patient selection, and the impact of regulatory risk minimization measures.
- Despite a higher proportion of serious classifications, upadacitinib-related VTE cases had a lower proportion of fatal outcomes compared to other JAKi and therapies, underscoring the complexity of assessing severity and outcomes in spontaneous reporting data.

## Introduction

The Janus kinase (JAK) family includes four intracellular non-receptor tyrosine kinases: JAK1, JAK2, JAK3, and tyrosine kinase 2 (TYK2) (1). Among them, JAK1 stands out for its ability to form heterodimers with the other three JAKs, playing a critical role in signaling pathways. JAK1 dysregulation is strongly associated with inflammation and severe autoimmune conditions (2), which has led to a growing interest from researchers in studying JAK1 inhibitors as therapeutic agents. One such inhibitor, upadacitinib, is an oral drug that selectively targets JAK1 by reversibly inhibiting its signaling pathway. After several studies, a robust safety profile between its risk and benefit was demonstrated concerning its administration in several diseases compared to placebo, offering a more tailored approach to managing immune-mediated conditions (3, 4). Upadacitinib was initially approved by regulatory agencies, including the Food and Drug Administration (FDA) (5) and the European Medicines Agency (EMA) (6), in 2019 for treating rheumatoid arthritis (RA). Therapeutic strategies for RA typically involve disease-modifying antirheumatic drugs (DMARDs), which comprise biologic agents (bDMARDs) and targeted synthetic agents (tsDMARDs), the latter including JAK inhibitors (JAKi), all aimed at controlling inflammation and halting disease progression (7, 8). Since then, its therapeutic indications have been expanded to include other chronic inflammatory diseases, such as dermatological and gastrointestinal conditions, including moderately to severely active Crohn’s disease (9), and more recently, giant cell arteritis (10). Notably, the FDA approved upadacitinib as the first oral treatment option for Crohn’s disease in patients with inadequate response or intolerance to tumor necrosis factor (TNF) blockers, highlighting its growing role in addressing unmet clinical needs across multiple inflammatory conditions (11, 12).

Despite the efficacy of JAK1 inhibitors to treat inflammatory and immune-mediated diseases, some concerns have been pointed out relating to their use, especially the increased risk of major adverse cardiovascular events (MACE), malignancy, mortality or occurrence of venous thromboembolic events (VTE) (13). In fact, in two studies carried out by the ORAL Surveillance trial (14, 15), with 4,362 included patients (1,455 receiving tofacitinib) and a median follow-up of 4.0 years, one of the JAKi, tofacitinib, was associated with an increase in MACE, cancer, VTE, pulmonary embolism and death. Opportunistic infections, such as herpes zoster or tuberculosis, were also associated with tofacitinib (14). Moreover, according to a study by Gouverneur et al. (16), which included 5,870 patients, 93.7% of whom were diagnosed with RA and treated with JAKi (tofacitinib and baricitinib), a total of 92 thromboembolic events were recorded. Of these, 41.3% were VTE, with a slight trend toward an increased risk, particularly in patients with pre-existing cardiovascular risk factors, such as hypertension and a history of smoking. After reviewing these results, the FDA warned about the risks of MACE, malignancies, and VTE with tofacitinib, restricting JAKi use to patients unresponsive to TNF inhibitors (TNFis). Based on comparable evidence, these restrictions were later extended to baricitinib and upadacitinib. Consequently, guidelines recommend a cautious approach when prescribing JAKi to patients at risk of thrombosis (17).

A recent trial involving 4,298 patients treated with upadacitinib across different indications found similar rates of MACE and VTE to those observed with other biologic treatments for RA, psoriatic arthritis, or ankylosing spondylitis, suggesting that upadacitinib does not entail increased risk compared to other therapies (18). From another perspective, a *post hoc* analysis pooling safety data from six phase III trials evaluated 3,209 patients treated with upadacitinib 15 mg daily (alone or in combination with methotrexate), adalimumab, or methotrexate monotherapy. The rates of MACE and VTE were higher among RA patients with increased cardiovascular risk but remained comparable across treatment groups. However, significantly increased rates of serious opportunistic infections and non-melanoma skin cancer were observed in upadacitinib-treated patients compared with comparators (19). In contrast, a systematic review and meta-analysis conducted by Partalidou et al. 20), which included 23 studies, found no statistically significant differences in the occurrence of MACE or DVT between JAKi and TNFi in patients with RA, highlighting the need for more conclusive evidence regarding the cardiovascular safety of JAKi.

Therefore, the present study aimed to address the ongoing controversy and evidence gaps regarding the safety profile of upadacitinib, particularly its potential association with VTE. Using post-marketing data from VigiBase, we conducted a disproportionality analysis to detect potential safety signals. The analysis was structured across three comparative levels: (i) a global comparison with all other medicines; (ii) a focused comparison with other second-line advanced therapies for RA, including both bDMARDs and tsDMARDs, as defined in the Portuguese clinical guidelines; and (iii) a targeted comparison with other JAKi. The aim was to explore the safety of upadacitinib both in a broad pharmacovigilance context and within its originally approved indication (RA), using a multilevel approach to provide insight into its risk profile across general and condition-specific settings.

## Materials and methods

### Source of data

This study is based on Individual Case Safety Reports (ICSRs) extracted from VigiBase, the World Health Organization (WHO) global database of ICSRs. Managed by the Uppsala Monitoring Centre (UMC), VigiBase compiles pharmacovigilance data from over 150 national drug regulatory authorities worldwide (21). The database is linked to standardized medical and drug classifications, including MedDRA (Medical Dictionary for Regulatory Activities) and WHODrug (WHO’s medicinal product dictionary), ensuring consistency in case coding, retrieval, and analysis. Reports in VigiBase originate from spontaneous reporting systems and regulatory submissions, which may vary in completeness and causality assessment, but collectively serve as a critical resource for global pharmacovigilance and signal detection.

### Study design and case selection

This study is a retrospective pharmacovigilance analysis evaluating the reporting pattern of VTE associated with upadacitinib in VigiBase. All ICSRs recorded up to February 20, 2025, were considered to define upadacitinib suspected cases (exposed cases) and several comparative groups (non-exposed cases), with no restrictions applied based on reporting country, patient demographics (age, gender), or report type. The study focused on VTE, identified using the Standardized MedDRA Query (SMQ) Narrow for “*Embolic and thrombotic events, venous*” (SMQ code: 20000084), which encompasses 102 Preferred Terms (PTs) related to venous thromboembolism. To minimize potential biases associated with spontaneous reporting, automated and manual verification excluded duplicate records and reports with incomplete case information.

We assessed the safety profile of upadacitinib for VTE by conducting a disproportionality analysis using post-marketing data from VigiBase, accessed via VigiLyze. The analysis was structured across three comparative levels. First, upadacitinib was compared with all other medicines in the database. Second, we restricted the comparison to other second-line advanced therapies for RA, as defined by the *Portuguese National Pharmacy and Therapeutics Committee*, including both bDMARDs (abatacept, rituximab, tocilizumab) and tsDMARDs, all of which are JAKi (baricitinib, tofacitinib, filgotinib). Third, we compared upadacitinib specifically with other tsDMARDs (i.e., JAKi: baricitinib, tofacitinib, filgotinib). These three comparator levels allowed for a multilevel evaluation of the safety profile of upadacitinib, both broadly and in relation to therapeutically and mechanistically similar agents.

### Data analysis and signal detection

A descriptive statistical analysis was initially carried out on the demographic and clinical characteristics of cases, including age, sex, country of primary source (grouped according to WHO geographic regions), year of VigiBase initial date, number of drugs per case (categorized as 1, 2–4, or ≥ 5 drugs reported), reported RA indication, number of AE per case, seriousness status and criteria, and fatal outcomes.

A disproportionality analysis was performed using two established signal detection methods for all drugs with at least three reports of the target AE: Reporting Odds Ratio (ROR) and Information Component (IC). Both methods were applied to each combination of the target drug (upadacitinib) and target AE (VTE) to identify potential safety signals. The ROR was calculated with a 95% confidence interval (95% CI), and a signal was considered statistically significant when the lower bound of the 95% CI exceeded 1, indicating a higher-than-expected reporting rate of VTE associated with upadacitinib relative to the comparator drugs. Based on a Bayesian statistical approach, the IC was also calculated with a 95% CI, and a signal was considered statistically significant when the lower bound of the CI 95% exceeded 0. Given that ROR is widely used but sensitive to sample size variations, potentially leading to false positives with low report counts, IC was used as a complementary approach to adjust for background reporting rates and reduce spurious associations (Supplementary Tables 1, 2).

To investigate potential patterns and predictors of disproportionality, subgroup analyses were conducted across several predefined categories (e.g., MedDRA PT, sex, age group, year of report, RA indication, Supplementary Table 3, and number of co-medicated drugs). These analyses aimed to characterize how the disproportionality of VTE reporting associated with upadacitinib may vary across relevant patient or reporting characteristics, which may represent potential confounding factors related to VTE occurrence and help discuss possible false-positive and false-negative findings.

This study was conducted using anonymized ICSRs from a spontaneous reporting database, and therefore, ethical approval and patient consent were not required. All analyses complied with international pharmacovigilance and pharmacoepidemiology standards, including those outlined by the WHO and the *European Network of Centres for Pharmacoepidemiology and Pharmacovigilance* (*ENCePP) Guide on Methodological Standards in Pharmacoepidemiology* (22). The study followed the *REporting of A Disproportionality Analysis for DrUg Safety Signal Detection Using Individual Case Safety Reports in PharmacoVigilance* (READUS-PV) guideline, ensuring methodological rigor in safety signal detection and adherence to pharmacovigilance best practices (23).

All statistical analyses were performed using R software (R Foundation for Statistical Computing, Vienna, Austria). Disproportionality analysis was implemented using the function “*da*” of the “*pvda*” package.^1^

## Results

### Descriptive analysis

**FIGURE 1:**
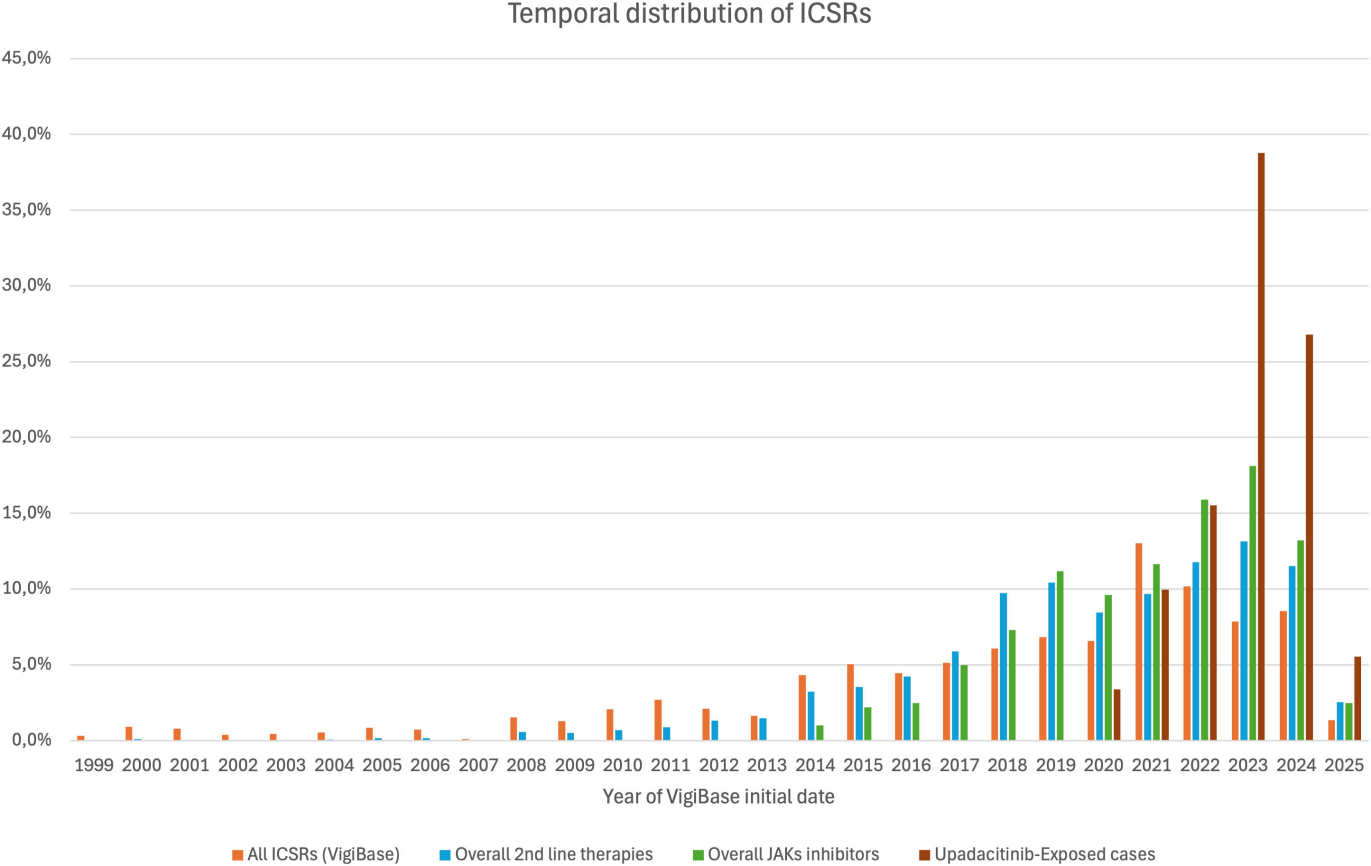
Temporal distribution of Individual Case Safety Reports (ICSRs) across different drug categories in VigiBase from 1999 to 2025. Each bar represents the proportion of ICSRs per year involving: any drug in VigiBase (orange), second-line therapies for rheumatoid arthritis (blue), JAK inhibitors (green), and upadacitinib (brown). Reports include any type of adverse event. Reporting begins later for drug classes authorized after 1999.

At the time of extraction, VigiBase included a total of 41,285,626 ICSRs, of which 552,319 (1.34%) were associated with at least one second-line therapy agent for RA under study. Among these, 228,997 ICSRs (41%) involved at least one JAKi, whether or not it included upadacitinib. Specifically, 62,257 ICSRs (27% of JAKi-related reports) identified upadacitinib as a suspected or interacting drug. Most cases involving upadacitinib were reported in 2023 (24,139; 39%), showing a temporal distribution similar to that of JAKi-related ICSRs and ICSRs involving any second-line therapy anti-RA drug (Figure 1 and Table 1).

**TABLE 1:** Characteristics of individual case safety reports (ICSRs) recorded in VigiBase, grouped by upadacitinib exposure and comparator groups.

Variable	All ICSRs (VigiBase)^1^	Upadacitinib-exposed cases^2^	Overall 2nd line therapies^3^	Other 2nd line therapies^4^	Other JAKs inhibitors^5^
	*N* = 41,285 626	*N* = 62,257 (0.15%)	*N* = 552,319 (1.34%)	*N* = 490,062 (1.19%)	*N* = 166,740 (0.40%)
**Drugs, n (%)**					
Abatacept	94,316 (0.2)	90 (0.1)	94,316 (17.1)	94,226 (19)	1,388 (0.8)
Baricitinib	21,529 (0.1)	95 (0.2)	21,529 (3.9)	21,434 (4.4)	21,434 (13)
Filgotinib	1,884 (0.3)	7 (< 0.1)	1,884 (0.3)	1,877 (0.4)	1,877 (1.1)
Rituximab	156,551 (0.4)	48 (< 0.1)	156,551 (28.3)	156,505 (32)	835 (0.5)
Tocilizumab	84,771 (0.2)	89 (0.1)	84,771 (15.3)	84,682 (17)	1,393 (0.8)
Tofacitinib	143,811 (0.3)	272 (0.4)	143,811 (26.0)	143,539 (29)	143,539 (86)
Upadacitinib	62,257 (0.2)	62,257 (100)	62,257 (11.3)	0 (0)	0 (0)
Number of cases involving a VTE, n (%)	269,520 (0.7)	894 (1.4)	5,579 (1.0)	4,685 (1.0)	2,262 (1.4)
**Number of suspect/interacting drugs per case, n (%)**					
1		58,518 (94)	439,506 (80)	380,988 (78)	148,950 (89)
2–4		3,531 (5.7)	75,056 (14)	71,525 (15)	14,848 (8.9)
5 +		208 (0.3)	37,757 (6.8)	37,549 (7.7)	2,942 (1.8)
Number of suspect/interacting drugs per case, Mean (SD)		1.09 (0.52)	1.62 (1.74)	1.69 (1.83)	1.24 (1.05)
**Number of co-medications per case, n (%)**					
1		43,690 (70)	318,502 (58)	274,812 (56)	112,779 (68)
2–4		12,313 (20)	114,177 (21)	101,864 (21)	30,948 (19)
5+		6,254 (10)	119,640 (22)	113,386 (23)	23,013 (14)
Number of co-medications per case, Mean (SD)		2.14 (2.99)	3.2 (4.1)	3.3 (4.2)	2.46 (3.36)
RA indication, n (%)		31,669 (51)	243,286 (44)	211,617 (43)	86,825 (52)
Upadacitinib with RA indication, n (%)		31,443 (51)	31,443 (5.7)	0 (0)	0 (0)
JAK with RA indication, n (%)		31,496 (51)	117,097 (21)	85,601 (17)	85,601 (51)
2nd line biologics with RA indication, n (%)		31,517 (51)	238,425 (43)	206,908 (42)	85,862 (51)
**Serious, n (%)**					
Yes	13,407,294 (37)	26,877 (43)	244,533 (45)	217,656 (45)	55,515 (33)
Unknown	4,643,487	5	10,604	10,599	342
**Seriousness criteria, n (%)**					
Death	1,453,679 (5.2)	1,172 (1.9)	25,091 (4.5)	23,919 (4.9)	3,756 (2.3)
Life threatening	654,727 (2.3)	345 (0.6)	10,715 (1.9)	10,370 (0.2)	1,510 (2.1)
Caused/prolonged hospitalization	4,678,310 (16.8)	9,510 (1.5)	88,715 (16.1)	79,205 (16.2)	18,391 (11)
Disabling/incapacitating	540,829 (1.9)	420 (0.7)	4,156 (0.8)	3,736 (0.8)	1,085 (0.7)
Congenital anomaly/birth defect	46,047 (0.2)	20 (< 0.1)	241 (< 0.1)	221 (<0.1)	35 (< 0.1)
Other medically important condition	7,061,422 (25.3)	19,199 (30.8)	160,772 (29.1)	141,573 (28.9)	41,585 (24.9)
**Reporter qualification, n (%)**					
Physician	10,746,449 (33.1)	12,647 (20)	151,568 (28.4)	138,921 (29)	34,329 (21)
Pharmacist	3,592,028 (11.1)	1,163 (1.9)	36,060 (6.7)	34,897 (7.4)	6,857 (4.2)
Other health professional	6,689,845 (20.6)	9,275 (15)	151,673 (28.4)	142,398 (30)	50,578 (31)
Lawyer	542,744 (1.7)	3 (< 0.1)	404 (0.1)	401 (0.1)	279 (0.2)
Consumer/non-health professional	13,204,411 (40.6)	40,196 (65)	220,014 (41.2)	179,818 (38)	82,289 (50)
Unknown	8,802,318	274	17,751	17,477	1,623
**Sex, n (%)**					
Female	23,463,668 (61)	42,227 (72)	367,775 (72)	325,353 (72)	126,929 (79)
Male	15,294,736 (39)	16,362 (28)	144,026 (28)	127,627 (28)	34,042 (21)
Unknown	2,527,222	3,668	40,518	36,848	5,769
**Patient age, n (%)**					
0–27 days	74,289 (0.2)	8 (< 0.1)	135 (< 0.1)	88 (< 0.1)	14 (< 0.1)
28 days to 23 months	873,166 (2.9)	2 (< 0.1)	535 (0.1)	500 (0.1)	50 (< 0.1)
2–11 years	1,160,797 (3.8)	31 (< 0.1)	3,819 (1)	3,788 (1.1)	350 (0.2)
12–17 years	916,128 (3.0)	546 (1.6)	4,695 (1.2)	4,148 (1.2)	717 (0.5)
18–44 years	8,915,168 (29.2)	7,402 (22)	60,113 (15)	52,677 (15)	19,300 (14)
45–64 years	10,088,413 (33.0)	15,863 (47)	175,429 (45)	159,458 (45)	68,623 (48)
65–74 years	4,832,220 (15.8)	6,820 (20)	93,939 (24)	87,077 (24)	35,873 (25)
75 + years	3,709,666 (12.1)	3,124 (9.2)	51,294 (13)	48,174 (14)	17,856 (13)
Unknown	10,715,779	28,461	162,360	133,918	23,957
**WHO region, n (%)**					
African, Eastern Mediterranean, South-East Asia, or Western Pacific Regions	11,368,331 (28)	2,324 (3.7)	67,391 (12)	65,067 (13)	8,932 (5.4)
European Region	10,221,680 (25)	14,300 (23)	119,556 (22)	105,256 (21)	22,291 (13)
Region of the Americas	19,695,615 (48)	45,633 (73)	365,372 (66)	319,739 (65)	135,517 (81)

^1^All ICSRs reported in the VigiBase database, regardless of suspected drug.

^2^ICSRs involving upadacitinib as a suspected drug.

^3^Comparator group for the disproportionality analysis: all second-line advanced therapies for RA (including abatacept, baricitinib, filgotinib, rituximab, tocilizumab, tofacitinib, and upadacitinib).

^4^Comparator group for the disproportionality analysis: all second-line advanced therapies for RA, excluding upadacitinib (including abatacept, baricitinib, filgotinib, rituximab, tocilizumab, and tofacitinib).

^5^Comparator group for the disproportionality analysis: all other JAK inhibitors (baricitinib, tofacitinib, filgotinib), excluding upadacitinib. Cases with “unknown” values were considered missing data; therefore, only valid percentages (i.e., excluding missing data) were reported in the descriptive analyses.

We identified 894 ICSRs in VigiBase in which upadacitinib was associated with a VTE, corresponding to 1.4% of ICSRs where upadacitinib was a suspected or interacting drug (exposed cases). This represents a similar prevalence of VTEs when compared with other JAKi-related ICSRs (2,262; 1.4%) but larger relative to other second-line therapies (4,684; 1.0%) and any other drugs (268,626; 0.7%).

Among alternative second-line therapies for RA management, the most reported in VigiBase were rituximab (156,505; 32%), tofacitinib (143,539; 29%), abatacept (94,226; 19%) and tocilizumab (84,682; 17%). Tofacitinib (143 539; 86%) was the most common JAKi reported among non-exposed cases within JAKi-related ICSRs. Most ICSRs documented only one suspected/interacting drug, regardless of the sample of cases considered. However, non-exposed groups tended to have a higher number of drugs per report. Just over half of the JAKi cases reported upadacitinib for a RA indication (upadacitinib: 31,443; 51%; other JAKi: 85,601; 51%), whereas the proportion of cases reporting other second-line therapies for an RA indication was lower (206,908; 42%). Upadacitinib (26,877; 43%) and other therapies (217,656; 45%) ICSRs were more frequently classified as serious when compared to ICSRs associated with other JAKi (55,515; 33%). However, the proportion of deaths in upadacitinib-related ICSRs (1,172; 4.4%) was the lowest among all groups, compared to other JAKi (3,756; 6.8%) and other therapies (23,919; 11%). This was also below the overall death proportion reported in VigiBase (1,453,679; 5.2%).

Most AE suspected to be related to a JAKi were reported by consumers or non-health professionals (upadacitinib: 40,196; 65%, other JAKi: 82,289; 50%). In contrast, reports associated with other second-line therapies were more evenly distributed, with physicians (138,921; 29%) and other health professionals (142,398; 30%) contributing substantially, indicating that reporting was not concentrated among consumers in these cases.

Compared to the overall ICSR dataset at VigiBase, reports involving a suspected or interacting second-line therapies for RA, whether a JAKi or not, were more frequently observed in women and patients aged over 44 years. While most ICSRs originated from the Region of the Americas (48%), a disproportionate number of reports involving upadacitinib (73%) came from this region.

### Disproportionality analysis

Upadacitinib was associated with a disproportionately higher reporting of VTE compared to all drugs in VigiBase (IC: 1.14 [1.04, 1.23]; ROR: 2.22 [2.08, 2.37]), as well as when explicitly compared to other second-line therapies used for RA (IC: 0.51 [0.41, 0.60]; ROR: 1.51 [1.40, 1.62]). However, when compared to other JAKi, upadacitinib demonstrates an overall comparable VTE risk (IC: 0.06 [−0.04, 0.15]; ROR: 1.06 [0.98, 1.15]). Notably, it is associated with significantly fewer VTE reports than baricitinib (IC: −0.60 [−0.69, −0.51]; ROR: 0.32 [0.29, 0.35]), but substantially more than tofacitinib (IC: 0.43 [0.34, 0.53]; ROR: 1.60 [1.47, 1.74]) (Table 2).

**TABLE 2:** Disproportionality analysis of venous thromboembolism (VTE) associated with upadacitinib, comparing it to different drug groups using reporting odds ratio (ROR) and information component (IC).

Comparator group	N_observed_ (= a)	N_expected_	N_drug_ (= a + b)	N_reaction_ (= a + c)	N_total_ (= a + b + c + d)	IC_025_	ROR_025_
All other drugs	894	406	62,257	269,520	41,285,626	1.04*	2.08*
All other 2nd-line agents^1^	894	629	62,257	5,579	552,319	0.41*	1.40*
All other JAKi^2^	894	858	62,257	3,156	228,997	−0.04	0.98
Abatacept	894	491	62,257	1,234	156,483	0.77*	3.55*
Baricitinib	894	1,354	62,257	1,820	83,691	−0.69	0.29
Filgotinib	894	912	62,257	939	64,134	−0.12	0.44
Rituximab	894	733	62,257	2,576	218,762	0.19*	1.24*
Tocilizumab	894	625	62,257	1,476	146,939	0.42*	1.90*
Tofacitinib	894	663	62,257	2,190	205,796	0.34*	1.47*

Notation for marginal totals (e.g., a + b, a + c, a + b + c + d) corresponds to the 2 × 2 contingency table structure presented in Supplementary Table 1.

* and bold font indicate values that exceed the established threshold for signal detection (IC_0,25_ > 0 or ROR_0,25_ >1), suggesting a potential safety signal.

^1^Includes abatacept, baricitinib, filgotinib, tofacitinib, rituximab, and tocilizumab.

^2^Includes baricitinib, filgotinib, and tofacitinib.

### Analysis by reaction

**FIGURE 2:**
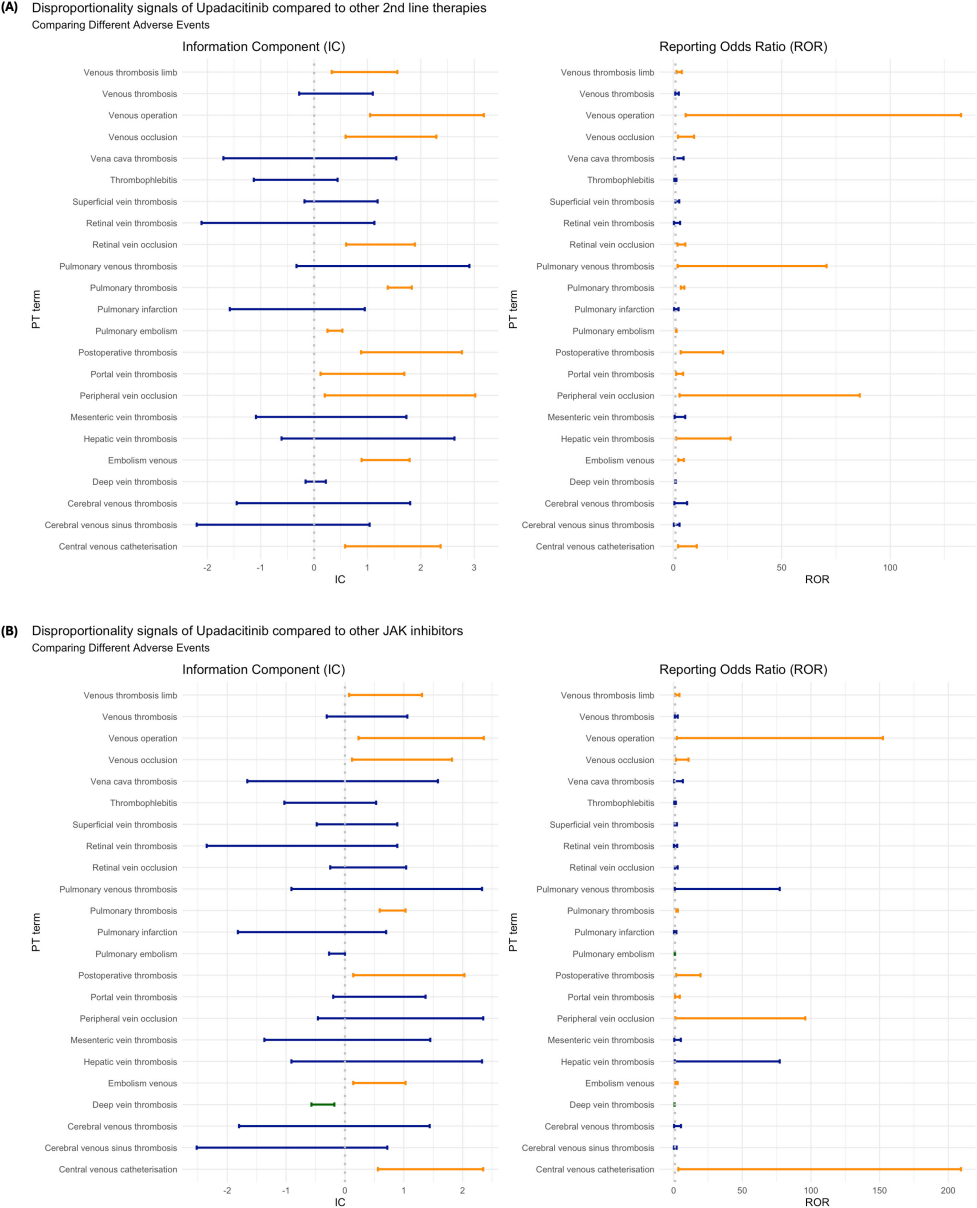
Disproportionality analysis of venous thromboembolism (VTE) associated with upadacitinib by Preferred Term (PT): **(A)** compared to other second-line therapies for rheumatoid arthritis; and **(B)** compared to other JAK inhibitors. Estimates are shown using the Information Component (IC) and Reporting Odds Ratio (ROR), each with 95% confidence intervals. Orange bars indicate statistically significant signals (IC_0,25_ > 0 or ROR_0,25_ > 1); blue bars indicate non-significant findings; green bars indicate inverse disproportionality (lower-than-expected reporting).

Despite an overall disproportionately higher reporting of VTE compared to other second-line therapies, this pattern varied when specific AE were considered (Figure 2A). Among the most reported PT terms (i.e., Pulmonary thrombosis, Pulmonary embolism, Deep vein thrombosis), upadacitinib showed an increased risk of pulmonary embolism (*n* = 420, IC: 0.4 [0.25, 0.53]; ROR: 1.37 [1.24, 1.52]) and pulmonary thrombosis (*n* = 160, IC: 1.62 [1.38, 1.83]; ROR: 4.21 [3.47, 5.10]), but no significantly different risk associated with deep vein thrombosis (DVT) (*n* = 219, IC: 0.03 [−0.16, 0.22]; ROR: 1.03 [0.89, 1.18]). Against other JAKi, upadacitinib was significantly less reported as being associated with deep vein thrombosis (IC: −0.37 [-0.57, −0.18]; ROR: 0.71 [0.61, 0.83]) and borderline significantly less pulmonary embolism (IC: −0.13 [−0.27, 0]; ROR: 0.88 [0.78, 0.99]), yet remained disproportionately more associated with pulmonary thrombosis (IC: 0.82 [0.59, 1.03]; ROR: 2.48 [2.00, 3.08]) (Figure 2B).

### Disproportionate signal analysis by other variables of interest

**FIGURE 3:**
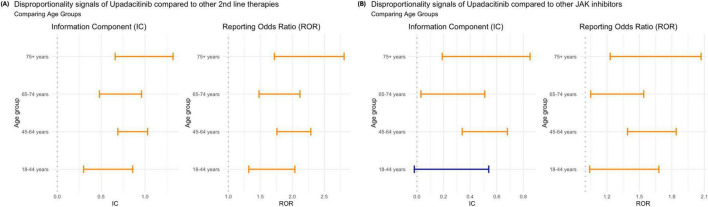
Disproportionality analysis of venous thromboembolism (VTE) associated with upadacitinib by age group: **(A)** compared to other second-line therapies for rheumatoid arthritis; and **(B)** compared to other JAK inhibitors. Estimates are shown using the Information Component (IC) and Reporting Odds Ratio (ROR), each with 95% confidence intervals. Orange bars indicate statistically significant signals (IC_0,25_ > 0 or ROR_0,25_ > 1; blue bars indicate non-significant findings.

Our results indicate no significant differences in VTE reporting trends across genders, regardless of the drug comparator group. Similarly, no notable variation was observed across age groups when upadacitinib was compared to other second-line therapies (Figure 3A). However, compared to other JAKi, nearly all age groups exhibited a disproportionately higher reporting of VTE associated with upadacitinib, contrasting with the overall findings that suggested no distinct safety profile. The only exception was among patients aged 18–44 years, for whom the reporting of upadacitinib-associated VTE did not reach statistical significance (IC: 0.27 [–0.02, 0.54]; ROR: 1.32 [1.04, 1.68]) (Figure 3B).

**FIGURE 4:**
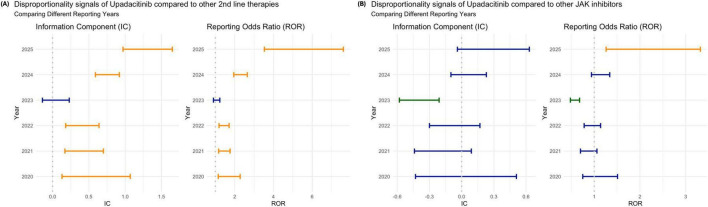
Disproportionality analysis of venous thromboembolism (VTE) associated with upadacitinib by reporting year: **(A)** compared to other second-line therapies for rheumatoid arthritis; and **(B)** compared to other JAK inhibitors. Estimates are shown using the Information Component (IC) and Reporting Odds Ratio (ROR), each with 95% confidence intervals. Orange bars indicate statistically significant signals (IC_0,25_ > 0 or ROR_0,25_ > 1; blue bars indicate non-significant findings; green bars indicate inverse disproportionality (lower-than-expected reporting).

In 2023, upadacitinib demonstrated a more favorable reporting pattern compared to other years. During this period, it was not associated with a significantly increased risk of VTE relative to other second-line therapies (IC: 0.05 [–0.14, 0.23]; ROR: 1.05 [0.90, 1.23]). Furthermore, it showed a significantly lower disproportionality in VTE reporting compared to other JAKi (IC: –0.38 [–0.58, –0.21]; ROR: 0.57 [0.48, 0.68]). In all other years, the safety profile of upadacitinib remained consistent with the overall findings observed across the entire study period (Figure 4).

**FIGURE 5:**
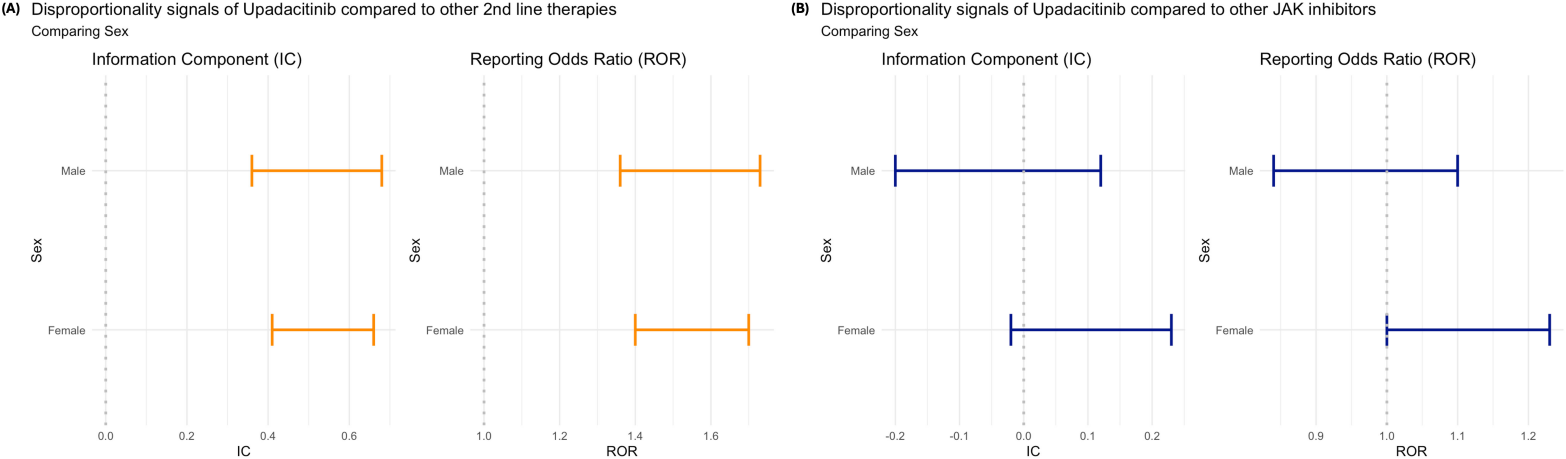
Disproportionality analysis of venous thromboembolism (VTE) associated with upadacitinib by patient sex: **(A)** compared to other second-line therapies for rheumatoid arthritis; and **(B)** compared to other JAK inhibitors. Estimates are shown using the Information Component (IC) and Reporting Odds Ratio (ROR), each with 95% confidence intervals. Orange bars indicate statistically significant signals (IC_0,25_ > 0 or ROR_0,25_ > 1; blue bars indicate non-significant findings.

When stratified by sex, the disproportionality analysis showed that, compared to other second-line therapies, upadacitinib was consistently associated with higher reporting of VTE in both male and female patients, with statistically significant disproportionality signals observed for men (IC: 0.50 [0.39, 0.60]; ROR: 1.54 [1.42, 1.68]) and women (IC: 0.55 [0.44, 0.65]; ROR: 1.50 [1.39, 1.63]) (Figure 5A). In contrast, when compared to other JAKi, no significant variation was observed between sexes, with both IC and ROR values close to the null (Figure 5B).

Subgroup analysis by the number of suspected or interacting drugs reinforced the disproportional signal of VTE occurrence associated with upadacitinib compared with other second-line therapies, as cases involving a single suspected or interacting drug remained statistically significant. When restricting the analysis to JAKi or other second-line therapies reported for an RA indication, the results were consistent with the overall findings: upadacitinib was associated with a disproportionately higher reporting of VTE compared with other second-line therapies, whereas, when compared with other JAKi, upadacitinib showed a comparable VTE reporting pattern (Supplementary Figures 1, 2).

## Discussion

To our knowledge, this is the first study to evaluate the global safety profile of upadacitinib in relation to VTE, using worldwide spontaneous ARD reports data from VigiBase, without temporal restrictions, through a structured multi-level disproportionality analysis of ICSR data to ensure comprehensive signal detection. The results indicate that upadacitinib is associated with a higher-than-expected reporting of VTE compared not only to all other drugs in VigiBase but also to other second-line therapies commonly used for RA, among which rituximab and tocilizumab showed a comparatively safer profile. Within the JAKi class, while the overall reporting pattern for upadacitinib was similar to other JAK inhibitors as a group, notable differences emerged at the individual drug level: upadacitinib was associated with fewer VTE reports than baricitinib but more than tofacitinib.

Importantly, the first-level comparison against all other medicines should be interpreted strictly as a global signal-screening step with limited clinical interpretability. Patients receiving upadacitinib typically have systemic inflammatory diseases with intrinsically elevated VTE risk – meta-analyses estimate ∼1.5–2.0-fold higher VTE risk in RA and ∼2–3-fold in IBD versus the general population – so indication-related confounding is expected at this level (24–26). Consistent with regulatory practices, disproportionality screening is an accepted first step in routine signal management but remains hypothesis-generating and requires contextualization with more specific comparators (27). Our second- and third-level analyses (advanced RA therapies and other JAKi) were designed precisely to mitigate this limitation and provide findings of greater clinical relevance. Notably, when the analysis was restricted to reports explicitly referring to RA, the results remained consistent with the overall findings, reinforcing the robustness of the observed disproportionality signal.

Mechanistically, Janus kinase/signal transducers and activators of transcription (JAK/STAT) signaling is intertwined with pathways of coagulation and immune-driven thrombosis. The JAK/STAT pathway plays a role in platelet production and activation, linking it to thrombosis pathophysiology (28). Inhibiting JAKs can simultaneously affect multiple cytokine networks—some that normally promote clot formation and others that protect against it. For example, blocking certain JAK-dependent cytokines that have anti-inflammatory and anti-thrombotic effects (such as IL-10 or type I interferons) might remove a protective brake on thrombosis, even if the drug also suppresses pro-thrombotic inflammatory signals (29). It has been hypothesized that selectively inhibiting one JAK isoform may upset the balance between pro- and anti-thrombotic signaling cascades, which could contribute to a prothrombotic tendency in susceptible patients (29). However, the precise biological mechanism by which JAKi might promote VTE remains uncertain, underscoring the need for further mechanistic studies (30). Understanding these drug-specific effects on coagulation pathways will be important to clarify why thrombotic events occur in some JAKi-treated patients and whether certain agents in this class carry lower thrombotic risk than others.

Notably, among biologics, rituximab and tocilizumab showed a comparatively safer VTE reporting profile, aligning with evidence that TNFα or IL-6 inhibitors may carry lower thrombotic risk (31). Within the JAK inhibitor class, upadacitinib’s overall reporting pattern was similar to the class as a whole, but important differences emerged at the individual drug level. Upadacitinib was associated with fewer VTE reports than baricitinib but more than tofacitinib, in line with recent pharmacovigilance analyses showing VTE, stroke, and ischemic heart disease events were more frequently reported with upadacitinib and baricitinib than with tofacitinib (32). This heterogeneity underscores that JAKi should not be viewed as a homogeneous group in terms of safety profile. Differences in JAK selectivity may partly explain the observed VTE reporting patterns. Baricitinib inhibits JAK1 and JAK2 and is uniquely associated with transient increases in platelet counts, possibly through JAK2-mediated thrombopoietin signaling, which could theoretically elevate thrombotic risk. In contrast, tofacitinib (JAK1/3) and upadacitinib (JAK1-selective) are generally associated with reductions in platelet counts (33, 34). Our data align with previous findings, indicating fewer VTE reports for upadacitinib than for baricitinib, but more than for tofacitinib. These differences highlight that JAK inhibitors should not be considered a homogeneous class when evaluating benefit–risk profiles. Despite its more selective mechanism, the disproportionality signal observed for upadacitinib suggests that its VTE risk cannot be disregarded. While this does not establish causality (35), it reinforces the need for ongoing monitoring and further investigation.

Another noteworthy observation was that upadacitinib-related VTE cases showed a lower proportion of fatal outcomes compared with other JAK inhibitors and second-line therapies. While this finding should be interpreted cautiously, several explanations may be considered. Differences in baseline patient characteristics, such as younger age or fewer comorbidities among upadacitinib users, could partly account for this pattern, as younger populations generally present lower VTE-related mortality risk. For instance, in a large Swedish cohort, patients with VTE and no comorbidities (Charlson Comorbidity Index = 0) had < 1% mortality at 3 months, whereas those with multimorbidity clusters (e.g., cardiometabolic or digestive diseases) showed markedly elevated VTE risks (adjusted ORs 3.44 and 4.35, respectively) (36). Previous pharmacovigilance data further support this interpretation: fatal ADRs represent just over 1% of all global reports, although not specific to VTE, and are disproportionately concentrated among older patients with comorbidities (37). Importantly, VTE-specific data indicate that case fatality differs substantially by event type, with 30-day mortality rates around 7–11% for pulmonary embolism compared with < 3% for isolated DVT (38, 39). These differences suggest that both patient characteristics and the relative distribution of PE versus DVT events may influence the observed lower fatality proportion in upadacitinib-related reports. Although causality cannot be established, this signal warrants further investigation in large, controlled real-world studies with adjustment for baseline thrombotic risk factors.

Therapeutic decision-making might be influenced in patients with a higher baseline VTE risk, such as those with a history of thrombosis, obesity, certain comorbidities, female sex, or prolonged immobility, by favoring treatments with a more favorable thrombotic profile when possible (31). Differences in AE reporting across disease states may also play a role. Upadacitinib is approved for multiple immune-mediated inflammatory conditions, including RA, axial spondyloarthritis (both radiographic and non-radiographic forms), psoriatic arthritis, giant cell arteritis, atopic dermatitis, and, more recently, inflammatory bowel disease (40–42). These indications differ in baseline thrombotic risk and patient profiles. For example, RA confers a twofold increased VTE risk compared to the general population, and active IBD is associated with an even higher risk (43). Such factors may influence reporting patterns and should be considered when interpreting upadacitinib’s safety signals. Furthermore, our subgroup findings indicate that stronger disproportionality signals were observed in cases with multiple concomitant drugs, suggesting that polypharmacy or combined immunosuppressive therapy—common in RA treatment—may contribute to the increased thrombotic risk reported with upadacitinib. This observation reinforces that the present data predominantly reflect RA treatment contexts, where co-administration of methotrexate, corticosteroids, or biologics is frequent. In contrast, in dermatological indications such as atopic dermatitis or alopecia areata, where JAK inhibitors are typically used as monotherapy and concomitant systemic immunosuppression is not allowed, the observed VTE disproportionality should not be directly extrapolated (13). These differences in therapeutic context and baseline thrombotic risk warrant careful consideration when generalizing safety signals across indications.

Interestingly, when upadacitinib was compared to the JAK inhibitor class overall (treating all JAKi as a single group), no significant disproportionality signal for VTE was detected in our analysis. However, as noted above, relevant differences emerged when comparing individual drugs, highlighting that the JAK inhibitor class is not monolithic in its risk profile. These findings reinforce that clinicians should avoid treating the JAKi group as homogeneous in clinical decision-making and instead consider the safety nuances of each agent. Consistent with this, regulators have extended findings from the ORAL Surveillance trial, which showed increased rates of serious cardiovascular events and VTE with tofacitinib in high-risk RA patients, to the entire JAK inhibitor class (14). Those results prompted the FDA and EMA to extend class-wide warnings to other JAKi, including upadacitinib, even though direct comparative evidence for the newer agents remains limited (44, 45). In fact, the FDA in 2021 required updated boxed warnings for upadacitinib and baricitinib, considering that they share mechanisms with tofacitinib and “may have similar risks,” and now recommends using JAKi only in patients who have an inadequate response to TNF blockers, with careful consideration of individual risk factors.

Real-world evidence on upadacitinib’s thromboembolic risk is still emerging. Several recent studies have investigated the link between JAKi and VTE risk. For example, a large multi-database study across 14 data sources in the USA, Europe, and Japan (post-marketing surveillance) showed a significantly elevated VTE incidence for baricitinib compared to TNF inhibitors (incidence rate ratio ≈1.51, 95% CI 1.10–2.08) (46). Data for upadacitinib in such studies remain scarce, as it is a newer agent, but so far, it has not demonstrated a clear signal in those settings. Notably, a French nationwide cohort study of RA patients initiating JAKi (primarily tofacitinib or baricitinib) versus adalimumab found no statistically significant increase in VTE risk (HR≈1.1, 95% CI 0.7–1.6), including among older patients with cardiovascular risk factors, providing some reassurance (47). However, VTE events are relatively rare, and such observational studies may lack power or have confounding by indication (as patients on JAKi might have had more severe disease activity, which itself heightens thrombotic risk). Therefore, the absence of a signal in some studies should be interpreted with caution. On balance, our disproportionality findings together with existing evidence suggest that the potential VTE risk with upadacitinib (and JAKi in general) should not be overlooked, especially in vulnerable populations, even as more data are needed for definitive conclusions.

When stratified by age, upadacitinib was associated with a significantly higher disproportionality signal for VTE across all age groups when compared to other JAKi, except in the cohort aged 18–44 years, where the signal was borderline. In contrast, this age-related pattern was not observed when comparing upadacitinib to non-JAKi therapies, suggesting that age-related variations in thrombotic risk may be more pertinent within the JAKi. These observations resonate with clinical insights from the literature (14–16). Rajasimhan et al. (48) noted that patients over 50 years of age—especially those with prior VTE or cardiovascular risk factors—are at higher risk of thromboembolic events under JAKi therapy. This aligns with safety warnings highlighting increased risk in this age group. Notably, the EMA recommends that tofacitinib should only be used in patients aged over 65 years when no suitable alternatives are available. Similarly, the ORAL Surveillance trial identified age as a key risk modifier, with those over 65 experiencing higher rates of major adverse cardiovascular events and VTE on tofacitinib (14). Consistent with this, regulatory authorities have issued specific warnings for older patients. Both the FDA and EMA now caution that JAKi should be used with particular care in individuals over 65 or those with other cardiovascular or malignancy risk factors, and recommend that such patients be treated with JAKi only if no suitable alternatives are available (44, 45). With the aging of society and the increasing number of elderly patients with chronic inflammatory diseases who may be candidates for JAKi, these findings are highly relevant. Elderly patients are inherently at increased risk for thrombosis (including DVT and pulmonary embolism) (49), as well as infections such as sepsis (50) and herpes zoster due to age-related frailty and comorbidities (51). Our results reinforce that upadacitinib (like other JAKi) should be used with great caution in older patients, especially during prolonged treatments. In practice, this means prescribers should thoroughly screen for cardiovascular and thrombotic risk factors and perform risk stratification before initiating a JAKi. Patients with multiple risk factors might be better managed with alternative therapies when possible (52). Taken together with existing evidence, these pharmacovigilance findings provide additional insights to help prescribers make informed treatment decisions for patients with an elevated baseline risk of thromboembolic events. In such cases, choosing a JAKi with a more favorable VTE risk–benefit profile, or considering a non-JAK therapy, may be advisable.

We also observed that reports involving the use of these agents are more frequent in women than in men. This discrepancy may be related to the epidemiology of the disease as well as to clinical and social factors. RA exhibits a markedly higher prevalence in women, with an estimated female-to-male ratio of approximately 3:1. Consequently, a greater proportion of women receive advanced therapies, including biologic agents, which is reflected in the higher number of associated safety reports (53, 54). Women present immunological particularities, more X chromosomes and differences in the hormonal influence on the immune system, which not only affect susceptibility to disease but also the response to biological treatments (53, 55). Our stratified analyses by event type and time yielded additional insights. DVT was less frequently associated with upadacitinib compared to other JAKi, whereas pulmonary embolism (or “pulmonary thrombosis”) showed a relatively higher reporting frequency with upadacitinib. In fact, among the most commonly reported specific VTE event terms for upadacitinib, pulmonary embolism signals were prominent and had narrower confidence intervals, suggesting a more robust signal. The reason for this disproportionality in event types is not entirely clear. One possibility is that pulmonary emboli, being acute and clinically serious events, are more likely to be recognized and reported in the context of a newer drug like upadacitinib, whereas milder DVT events might be underreported or not immediately linked to the drug. It is notable that in the ORAL Surveillance trial and other analyses, the excess risk observed with JAKi was largely driven by pulmonary embolism events (14). High-dose tofacitinib, for example, was associated with a dose-dependent increase in pulmonary embolism risk (up to sixfold at 10 mg BID) compared to TNF blockers, even though differences in simple DVT were less pronounced (31). A plausible explanation is that JAKi may precipitate central or severe thrombotic events such as pulmonary embolism in susceptible patients, possibly through complex interactions between systemic inflammation, endothelial dysfunction, and coagulation pathways that are modulated by JAK-STAT signaling. Inflammation itself is a recognized risk factor for thrombosis in RA, and JAKi, while reducing pro-inflammatory cytokines, may also interfere with protective cytokines that regulate vascular homeostasis, potentially shifting the hemostatic balance toward thrombosis in certain contexts (56). However, the precise biological mechanisms underlying this potential prothrombotic tendency remain uncertain, warranting further mechanistic and clinical investigation. Meanwhile, clinicians should remain vigilant for early signs of pulmonary embolism (e.g., acute dyspnea, pleuritic chest pain, unexplained tachycardia) in patients receiving upadacitinib, given the importance of prompt detection and intervention in improving outcomes.

Additionally, we also observed that in 2023, upadacitinib showed a more favorable disproportionality profile (i.e., a lower reporting ratio for VTE) compared to other JAKi, coinciding with the highest volume of upadacitinib-related reports. This trend could reflect broader use of upadacitinib across indications and regions, as well as shifts in reporting patterns. Upadacitinib’s usage expanded significantly in 2022–2023, with approvals for atopic dermatitis and Crohn’s disease, leading to a large increase in the number of treated patients and ICSRs submitted (35). These new indications also change the user population, introducing patients with different baseline VTE risks, which may contribute to the observed signal attenuation. As upadacitinib’s patient exposure has grown, AE may now represent a smaller share of total reports, diluting disproportionality signals. Additionally, post-2021 prescribing became more selective, with JAKi increasingly avoided in high VTE-risk patients, possibly lowering VTE incidence in 2023 compared to earlier periods or to drugs like baricitinib. Given that the therapeutic indication is often missing in ICSRs, future studies, including time-series analyses aligned with approval dates for new indications, could help clarify evolving safety patterns. Continued monitoring will be needed to determine if this favorable trend for upadacitinib persists with broader use.

Overall, our findings underscore the need for further targeted research to clarify the VTE risk associated with upadacitinib and other JAKi. Large-scale real-world cohort or active surveillance studies with appropriate comparators and baseline risk adjustment are warranted to quantify absolute and relative VTE risks. For instance, a multi-database study found that baricitinib users had a ∼50% higher incidence of VTE compared to TNF inhibitors (IRR = 1.51; 95% CI 1.10–2.08) (31), while systematic reviews of RCTs report an increased odds of VTE with JAKi, reaching OR2.17 (95% CI 1.16–4.05) in analyses restricted to trials with ≥ 12 months follow-up (30). It remains uncertain whether the observed signals are driven primarily by pharmacologic effects – given that JAKi can interfere with platelet activation and endothelial function – or by patient characteristics such as age, comorbid cardiovascular risks, and disease activity, or a combination of both (57).

### Limitations

The present study has several limitations that should be considered when interpreting the findings. Using VigiBase, a spontaneous reporting system, precludes the estimation of the true incidence or prevalence of VTE among patients exposed to upadacitinib and other JAKi, as underreporting, differential reporting by country and healthcare setting, and potential stimulated reporting following regulatory alerts or media coverage can introduce bias and affect signal detection sensitivity (58). Disproportionality analysis is a well-established tool for early signal detection, but it is inherently hypothesis-generating and can never serve as a confirmatory method. Its results cannot establish causality and are limited by the quality and completeness of ICSRs, which often lack detailed data on disease severity, indication, treatment duration, dosage, baseline thrombotic risk factors, patient comorbidities, and concomitant medications such as corticosteroids or NSAIDs, all of which may modulate VTE risk (59). The timing of VTE occurrence relative to drug initiation is frequently missing, complicating temporal association assessments. In addition, we could not stratify our analysis by therapeutic indication (e.g., RA, atopic dermatitis, Crohn’s disease), despite evidence that underlying disease activity and inflammation may influence thrombotic risk profiles across conditions for which upadacitinib is prescribed. This limitation results from the frequent absence of therapeutic indication in ICSRs within VigiBase, which prevented reliable allocation of cases to specific clinical contexts. Consequently, our findings may be subject to indication bias, as patients receiving upadacitinib for different conditions may present heterogeneous baseline risks for VTE. We explicitly acknowledge this and emphasize that our multilevel comparator design (including RA-specific comparators) was intended to mitigate, though not completely eliminate, this potential source of bias. Differences in healthcare systems, pharmacovigilance infrastructure, and prescribing practices across the over 140 countries contributing to VigiBase can also affect reporting patterns, and while our analysis included all available data without temporal restrictions to maximize sensitivity, changes in prescribing patterns and regulatory risk minimization measures in recent years, such as the 2022 EMA recommendations for JAKi, may have influenced reporting rates over time, potentially attenuating disproportionality signals. Additionally, the comparator groups used (all drugs, second-line therapies for RA, and other JAKi) provided a multi-level perspective but residual confounding is likely, as we could not adjust for baseline thrombotic risk factors and comorbidities, which may differ between treatment groups and substantially influence VTE risk. This limitation restricts causal inference, and our results should be interpreted strictly as hypothesis-generating signals rather than evidence of a definitive causal relationship. Despite these limitations, the use of VigiBase enables global, real-world monitoring of rare but serious AE such as VTE across diverse populations, supporting pharmacovigilance efforts and generating hypotheses for targeted active surveillance and comparative cohort studies that can better delineate the absolute and relative risks associated with upadacitinib and other JAKi to guide safer and personalized prescribing strategies.

## Conclusion

This global real-world pharmacovigilance study provides important insight into the safety profile of upadacitinib, particularly its potential association with thromboembolic events, and highlights nuances within the JAKi class that are relevant for clinical decision-making. These findings, together with prior evidence, may assist prescribers in stratifying thromboembolic risk and selecting appropriate treatment for patients with RA and other immune-mediated conditions, balancing effectiveness with individual safety considerations. Future research should prioritize robust, long-term comparative pharmacoepidemiological studies using real-world cohorts to better clarify absolute and relative risks, establish causal relationships, and inform the development of practical risk stratification tools for clinical use. Ongoing pharmacovigilance will remain crucial to optimize the safe use of JAKi and to support evidence-informed, individualized treatment decisions that adapt to emerging safety data.

## Data Availability

Publicly available datasets were analyzed in this study. This data can be found here: VigiBase.

## Footnotes

1
https://cran.r-project.org/web/packages/pvda/vignettes/Disproportionality_analysis.html
