# Supplementary material for: Post-marketing surveillance of upadacitinib: multilevel analysis of venous thromboembolism reporting in global data and rheumatoid arthritis
Source: Front Med (Lausanne). 2025 Dec 1;12:1683751. doi: 10.3389/fmed.2025.1683751 (PMC12702925; doi:10.3389/fmed.2025.1683751)

**Supplementary Material**

**Table S1.** Structure of the contingency table used in disproportionality analyses for upadacitinib-associated venous thromboembolism (VTE).

|  | **VTE cases (target AE)** | **Non-VTE cases** | **Total** |
| --- | --- | --- | --- |
| Upadacitinib | a | b | a+b |
| Comparator group**^1^** | c | d | c+d |
| Total | a+c | b+d | n=a+b+c+d |

AE: adverse events; VTE: venous thromboembolism

**^1^** Comparator groups used in separate analyses: all other drugs in VigiBase; all other second-line therapies for rheumatoid arthritis; and all other JAK inhibitors for rheumatoid arthritis.

**Table S2.** Algorithms, formulas and signal detection thresholds used in the disproportionality analysis.

| **Algorithms** | **Formula** | **Threshold** |
| --- | --- | --- |
| ROR | ROR = (a × d) / (b × c) | ROR_0,25_ >1, a≥3 |
|  | 95% CI = e^[ln(ROR) ± 1.96 × √(1/a + 1/b + 1/c + 1/d)]^ |  |
| IC | IC = log₂[(a × N) / ((a + b)(a + c))] | IC_0,25_ >0, a≥3 |
|  | 95% CI = E(IC) ± 2 × √V(IC) |  |

95% CI: 95% confidence interval; IC: information component; IC₀.₂₅: lower limit of the 95% CI of the IC; N: total number of Individual Case Safety Reports in the database; ROR: reporting odds ratio; V(IC): variance of the IC.

**Table S3.** List of the 83 therapeutic indication descriptors retrieved from VigiBase and used to identify and define cases classified under the rheumatoid arthritis indication group.

| **#** | **Indication descriptor** | **Observations** |
| --- | --- | --- |
| 1 | ar |  |
| 2 | AR |  |
| 3 | AR LRS |  |
| 4 | AR RICARTE SOTO |  |
| 5 | AR refractaria |  |
| 6 | ARTRITIS REMAUTOIDEA |  |
| 7 | ARTRITIS REUMATICA |  |
| 8 | ARTRITIS REUMATOIDE LEY RICARTE SOTO |  |
| 9 | ARTRITIS REUMATOIDE REFRACTARIA |  |
| 10 | ARTRITIS REUMATOIDE REFRACTARIA - LRS |  |
| 11 | ARTRITIS REUMATOIDE REFRACTARIA A TRATAMIENTO |  |
| 12 | ARTRITIS REUMATOIDE SEROPOSITIVA |  |
| 13 | ARTRITIS REUMATOIDEA |  |
| 14 | ARTRITIS REUMATOIDEA REFRACTARIA |  |
| 15 | ARTRITIS REUMATOISE |  |
| 16 | ARTRITIS REUMATOOIDE |  |
| 17 | Arthritis rheumatoid |  |
| 18 | Arthritis rheumatoid aggravated |  |
| 19 | Artritis Rematuoidea |  |
| 20 | Artritis Remautoidea |  |
| 21 | Artritis Reumatoide Activa |  |
| 22 | Artritis Reumatoide Idiopática Sistémica |  |
| 23 | Artritis Reumatoide Refractaria |  |
| 24 | Artritis Reumatoide Refractaria, Ley Ricarte Soto |  |
| 25 | Artritis Reumatoide refractaria |  |
| 26 | Artritis Reumatoidea |  |
| 27 | Artritis Reumatoidea Refractaria |  |
| 28 | Artritis Reumatoride refractaria |  |
| 29 | Artritis reumatoide reflactaria |  |
| 30 | Artritis reumatoide refractaria |  |
| 31 | Artritis reumatoidea |  |
| 32 | Artritis reumatoidea refractaria |  |
| 33 | Artritis reumatoidea severa refractaria |  |
| 34 | Arttritis reumatoide |  |
| 35 | M05 | ICD 10 code for “Rheumatoid arthritis with rheumatoid factor” |
| 36 | M05.9 | ICD 10 code for “Rheumatoid arthritis with rheumatoid factor, unspecified” |
| 37 | M06.9 | ICD 10 code for “Rheumatoid arthritis, unspecified” |
| 38 | Malignant rheumatoid arthritis |  |
| 39 | Other rheumatoid arthritis |  |
| 40 | Other rheumatoid arthritis: site unspecified |  |
| 41 | Palindromic rheumatoid arthritis |  |
| 42 | Progression of rheumatoid arthritis |  |
| 43 | Pulmonary rheumatoid nodule |  |
| 44 | Rhematoid arthritis |  |
| 45 | Rheumatic arthritis acute |  |
| 46 | Rheumatoid arthiritis |  |
| 47 | Rheumatoid arthritis |  |
| 48 | Rheumatoid arthritis & other inflammatory polyarthropaties |  |
| 49 | Rheumatoid arthritis aggravated |  |
| 50 | Rheumatoid arthritis flare up |  |
| 51 | Rheumatoid arthritis in remission |  |
| 52 | Rheumatoid arthritis relapse |  |
| 53 | Rheumatoid arthritis, unspecified |  |
| 54 | Rheumatoid arthritis-associated interstitial lung disease |  |
| 55 | Rheumatoid arthropathy |  |
| 56 | Rheumatoid bursitis |  |
| 57 | Rheumatoid lung |  |
| 58 | Rheumatoid nodule |  |
| 59 | Rheumatoid polyarthritis |  |
| 60 | Rheumatoid scleritis |  |
| 61 | Rheumatoid vasculitis |  |
| 62 | Seronegative rheumatoid arthritis |  |
| 63 | Seropositive RA |  |
| 64 | Seropositive rheumatoid arthritis |  |
| 65 | Seropositive rheumatoid arthritis, unspecified |  |
| 66 | Systemic rheumatoid arthritis |  |
| 67 | TRATAMIENTO ARTRITIS REUMATOIDE |  |
| 68 | artritis reuatoidea |  |
| 69 | artritis reumatoide (AR) |  |
| 70 | artritis reumatoide activa |  |
| 71 | artritis reumatoide refractaria |  |
| 72 | artritis reumatoide refractaria a tratamiento habi |  |
| 73 | artritis reumatoidea |  |
| 74 | artritis reumatoidea refractaria |  |
| 75 | artritis reumatoides activa |  |
| 76 | artritris reumatoidea |  |
| 77 | artrtits reumatoide |  |
| 78 | atritis reumatoide |  |
| 79 | 类风湿关节炎并发神经病 | Rheumatoid arthritis with neuropathy |
| 80 | 类风湿性关节炎 | Rheumatoid arthritis |
| 81 | 类风湿病 | Rheumatoid disease |
| 82 | 累及全身的类风湿性关节炎 | Systemic involvement Rheumatoid arthritis |
| 83 | 风湿性关节 | Rheumatoid arthritis |

**Figure S1.** Disproportionality analysis of venous thromboembolism (VTE) associated with upadacitinib by therapeutic indication, as reported in individual case safety reports: **(A)** compared to other second-line therapies for rheumatoid arthritis; and **(B)** compared to other JAK inhibitors. Estimates are shown using the Information Component (IC) and Reporting Odds Ratio (ROR), each with 95% confidence intervals. Orange bars indicate statistically significant signals (IC₀,₂₅ > 0 or ROR₀,₂₅ > 1); blue bars indicate non-significant findings; green bars indicate inverse disproportionality (lower-than-expected reporting).


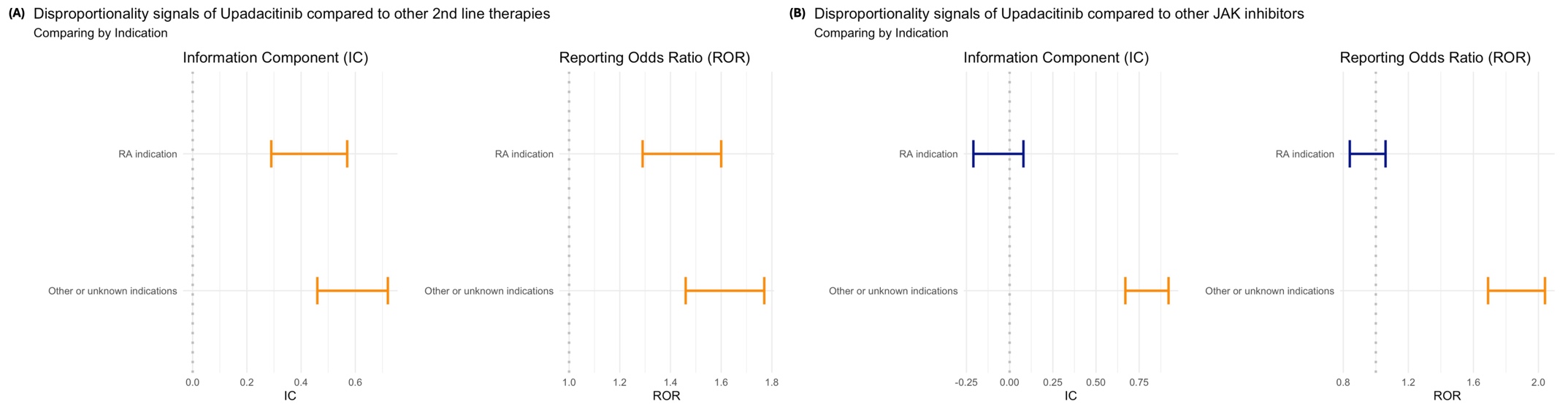


**Figure S2.** Disproportionality analysis of venous thromboembolism (VTE) associated with upadacitinib by the number of suspected or interacting drugs, as reported in individual case safety reports: **(A)** compared to other second-line therapies for rheumatoid arthritis; and **(B)** compared to other JAK inhibitors. Estimates are shown using the Information Component (IC) and Reporting Odds Ratio (ROR), each with 95% confidence intervals. Orange bars indicate statistically significant signals (IC₀,₂₅ > 0 or ROR₀,₂₅ > 1); blue bars indicate non-significant findings; green bars indicate inverse disproportionality (lower-than-expected reporting).


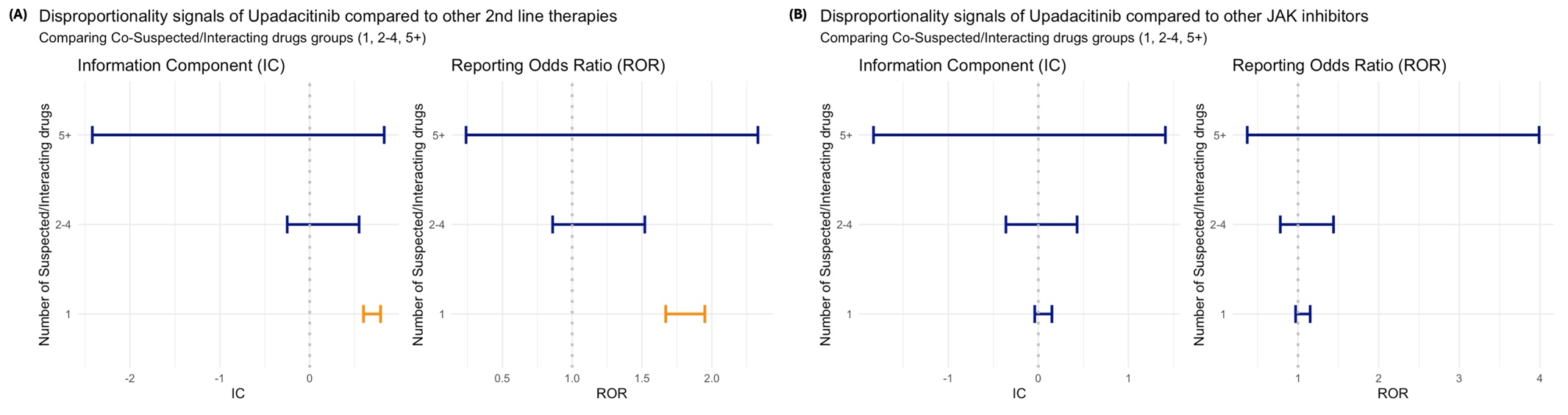

Supplement: Supplementary file 1 [file Supplementary_file_1.docx]
